# Potassium Fertilization Reduces the Severity of Leaf Spot Complex and Mosaic in *Megathyrsus maximus* Cultivars

**DOI:** 10.3390/plants15081244

**Published:** 2026-04-17

**Authors:** Emmanuel Lievio de Lima Véras, Gelson dos Santos Difante, Denise Baptaglin Montagner, Alexandre Romeiro de Araújo, Manuel Claudio Motta Macedo, Gustavo de Faria Theodoro, Carolina Marques Costa Araújo, Antônio Leandro Chaves Gurgel, Gabriela Oliveira de Aquino Monteiro, Jéssica Gomes Rodrigues, Marislayne de Gusmão Pereira, Juliana Caroline Santos Santana

**Affiliations:** 1 Department of Veterinary Medicine, Anclivepa College, Natal 59032-445, Rio Grande do Norte, Brazil; emmanuel.veras@hotmail.com; 2 Faculty of Veterinary Medicine and Animal Science, Federal University of Mato Grosso do Sul, Campo Grande 79070-900, Mato Grosso do Sul, Brazil; gelson.difante@ufms.br (G.d.S.D.); gustavo.theodoro@ufms.br (G.d.F.T.); marislayne@outlook.com (M.d.G.P.); 3 Embrapa Beef Cattle, Brazilian Agricultural Research Corporation, Campo Grande 79106-550, Mato Grosso do Sul, Brazil; denise.montagner@embrapa.br (D.B.M.); alexandre.araujo@embrapa.br (A.R.d.A.); manuel.macedo@embrapa.br (M.C.M.M.); 4 Department of Animal Science, Federal University of Grande Dourados, Dourados 79825-070, Mato Grosso of Sul, Brazil; carolinaufgd@hotmail.com; 5 Campus Professora Cinobelina Elvas, Federal University of Piaui, Bom Jesus 64900-000, Piaui, Brazil; antonio.gurgel@ufpi.edu.br; 6 Department of Animal Science, São Paulo State University “Júlio de Mesquita Filho”—UNESP, São Paulo 01049-010, Jaboticabal, Brazil; gabrielaoliveiraaquino@gmail.com; 7 Graduate Program in Animal Production, Federal University of Rio Grande do Norte, Macaíba 59280-000, Rio Grande of Norte, Brazil; jukrol_@hotmail.com

**Keywords:** brown spot, *Panicum maximum*, management

## Abstract

The objective of this study was to evaluate the effects of potassium (K) on disease severity and the chemical composition of *Megathyrsus maximus* cultivars. The experiment was conducted in a randomized block design in a 6 × 4 factorial arrangement, consisting of six *Megathyrsus maximus* cultivars (Massai, Mombaça, Tamani, Tanzânia, Quênia, and Zuri) and four K doses (0, 205, 410, and 820 mg dm^−3^). The severity of the leaf spot complex, caused by Bipolaris maydis and B. yamadae, was assessed using a diagrammatic scale. A significant interaction between K doses and cultivars was observed for all evaluated diseases. At K doses of 0 and 205 mg dm^−3^, the Tanzânia cultivar showed lower leaf spot severity compared with the other cultivars, whereas at higher doses, no disease symptoms were observed in any cultivar. The area under the disease progress curve (AUDPC) for mosaic followed a linear model only for the Tanzânia cultivar, whereas quadratic regression models best described the response for the remaining cultivars, with maximum mosaic severity values of 67.74% for Quênia, 72.34% for Mombaça, 76.99% for Zuri, 74.88% for Massai, and 68.93% for Tamani. Increasing K doses reduced the severity of both the leaf spot complex and mosaic. However, the leaf spot complex did not affect the nutritional value of the evaluated cultivars.

## 1. Introduction

Forage cultivars belonging to the species *Megathyrsus maximus* (syn. *Panicum maximum*) are widely used in livestock production systems based on cultivated pastures due to their high productivity and forage quality, characterized by abundant leaf production and good adaptation to a wide range of climates and soil conditions, thereby contributing to increased animal productivity [1].

These grasses exhibit facultative apomictic reproduction, resulting in a high proportion of asexual seed reproduction [2]. This reproductive strategy often leads to extensive clonal monocultures with low genetic variability, increasing genetic vulnerability and facilitating the occurrence and spread of diseases [2,3]. This vulnerability is further aggravated by reports indicating increased disease incidence and severity under conditions of soil nutrient deficiency [4,5,6]. Improvements in soil fertility enhance plant resistance, partly due to increased cell density in the leaf epidermis, which acts as a physical barrier against pathogen penetration and pest attack [7].

Among plant cell wall constituents that contribute to resistance against pathogen entry, cutin, complex phenolic compounds, and carbohydrates are particularly important [8]. In this context, adequate potassium (K) nutrition plays a central role, as high K concentrations activate more than 60 enzymes involved in carbohydrate synthesis and metabolic processes [9]. Furthermore, elevated K levels stimulate the production of high molecular weight compounds, such as proteins, starch, and cellulose, which are fundamental to plant defense mechanisms [10,11]. Conversely, potassium reduces the accumulation of soluble sugars and organic acids that favor pathogen development and insect feeding [9]. Among diseases affecting forage grasses, the foliar spot complex is particularly important, resulting from the simultaneous occurrence of leaf spot [3] and brown spot [12]. Another relevant disease of viral origin affecting these grasses is mosaic [13,14].

Leaf spot is caused by *Bipolaris maydis*, first reported in 2003 as the causal agent of leaf spot in the Tanzânia cultivar, which is considered highly susceptible to the disease [15]. Subsequent reports identified the pathogen in other genotypes of *Panicum, Brachiaria* spp., *Paspalum* spp., and *Pennisetum* [15,16]. Infected plants initially develop small, elliptical brown lesions that expand as the disease progresses, sometimes forming elongated necrotic areas [17]. More recently, *Bipolaris yamadae* has been associated with brown spot in *P. maximum* cv. Tamani in Brazil [12]. This disease is characterized by small, brownish, elliptical necrotic spots that, under high temperature and humidity conditions, coalesce and lead to significant loss of photosynthetically active leaf area, particularly in older leaves.

The mosaic virus affecting forage grasses belongs to the genus *Potyvirus* (family *Potyviridae*). It was first described in Australia as dwarf corn mosaic virus and later identified in Brazil as a strain of sugarcane mosaic virus, receiving specific nomenclature from EMBRAPA Dairy Cattle as *Johnsongrass mosaic virus* (JGMV) [18]. Symptomatic leaves display chlorotic streaks and spots, sometimes accompanied by necrosis, resulting in reduced forage productivity. Transmission occurs mechanically and not via seeds [13].

The relationship between potassium nutrition and plant metabolism, growth, and interactions with other nutrients in the soil–plant system provides several pathways through which K availability may influence plant resistance or susceptibility to diseases [19]. Therefore, the hypothesis tested in this study was that increasing soil K supply reduces the severity of foliar spot complex and mosaic diseases, consequently influencing plant nutritional value. Thus, the objective of this study was to evaluate the effects of potassium fertilization on disease severity and its impact on the nutritional value of different *Panicum maximum* cultivars.

## 2. Results

In the descriptive statistics of the diseases and chemical composition of the evaluated forages (Table 1), mean severity values (%) of 26.72 ± 26.01 were observed for the leaf spot complex, 69.52 ± 10.12 for mosaic, 91.65 ± 1.29 for dry matter (%), 5.56 ± 1.29 for mineral matter (%), 7.61 ± 1.61 for crude protein (%), 71.18 ± 1.61 for NDF (%), 38.93 ± 2.09 for ADF (%), 43.95 ± 5.26 for DIGMO (%), 3.52 ± 0.55 for lignin (%), 33.95 ± 1.94 for cellulose (%), and 1.61 ± 1.03 for silica (%). The highest coefficients of variation were observed for leaf spot severity and silica content among *Panicum maximum* cultivars. The remaining parameters showed low variation (Table 1).

There was an interaction between potassium dose and cultivar for the severity of the leaf spot complex (*p* = 0.0001) and mosaic (*p* = 0.0001) in *Panicum maximum* cultivars (Table 2). The application of increasing K doses to the soil reduced the severity of the leaf spot complex in all evaluated cultivars. In the absence of potassium fertilization, greater severity of the leaf spot complex was observed in cv. Massai, lower severity in Tanzânia, and intermediate severity in the other cultivars. At the K dose of 205 mg dm^−3^, greater severity was observed in cv. Mombaça, lower severity in Tanzânia and Zuri, and intermediate values in the remaining cultivars. At K doses of 410 and 820 mg dm^−3^, the evaluated forages did not show disease symptoms (Table 2).

Mosaic severity data fitted an increasing linear regression model only for the Tanzânia cultivar. For the remaining cultivars, mosaic severity was best explained by quadratic regression models. Based on these models, the estimated K dose corresponding to the maximum mosaic severity (inflection point) differed among cultivars, occurring at Quênia (530 mg K dm^−3^; 67.74%); Mombaça (307 mg K dm^−3^; 72.34%); Zuri (451.25 mg K dm^−3^; 76.99%); Massai (416 mg K dm^−3^; 74.88%); and Tamani (458.75 mg K dm^−3^; 68.93%).

At K doses of 0 and 205 mg dm^−3^, no differences were observed among cultivars in the expression of mosaic virus symptoms. However, at the K dose of 410 mg dm^−3^, the Tanzânia cultivar exhibited greater severity than the Quênia and Tamani cultivars. At the highest evaluated dose (820 mg dm^−3^), the Tanzânia cultivar showed the greatest mosaic severity (Table 2).

The observed severity of the leaf spot complex was correlated with DM, NDF, MM, and CEL contents, and positively correlated with CP, while no significant associations were observed with ADF, DIGOM, or LIG (Table 3). Mosaic severity was correlated with DM, CP, and MM contents and positively correlated with NDF and cellulose; however, no significant associations were observed with ADF, DIGOM, LIG, or silica (Table 3).

## 3. Discussion

**Figure 1:**
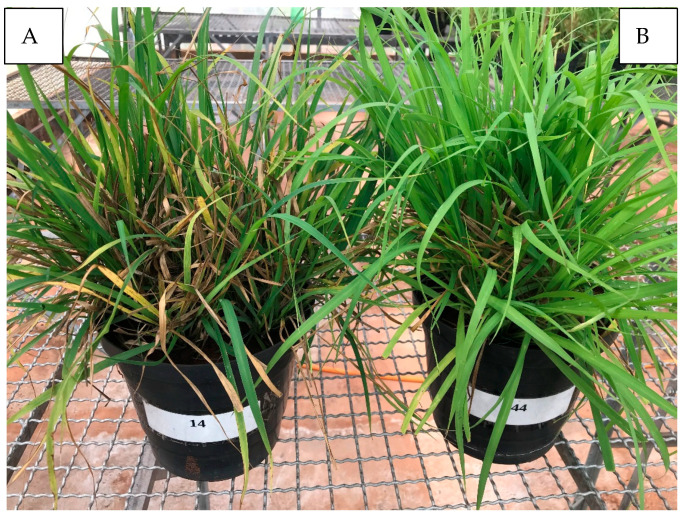
Effect of potassium fertilization on the severity of the brown spot/*Bipolaris maydis* leaf spot complex in the Massai cultivar. (**A**) control, dose 0; (**B**) 205 mg dm^−3^ of K.

**Figure 2:**
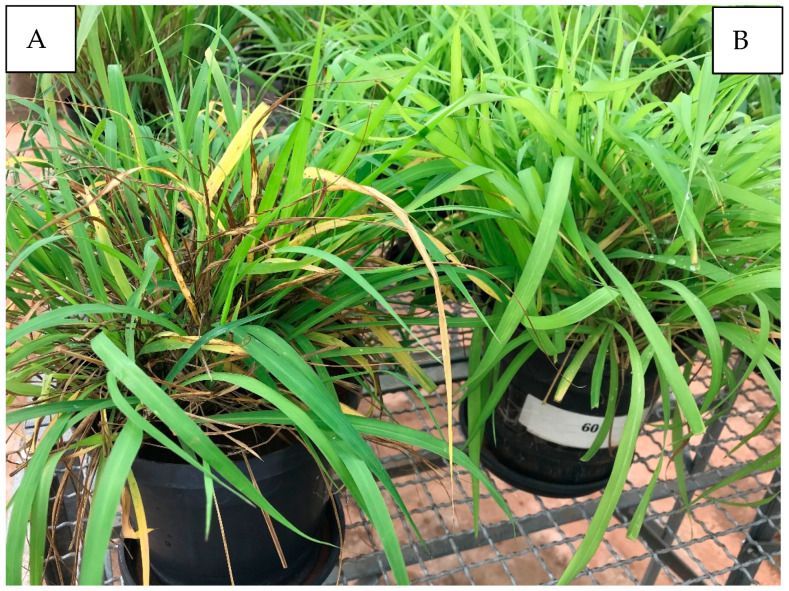
Effect of potassium fertilization on the severity of the brown spot/*Bipolaris maydis* leaf spot complex in the BRS Tamani cultivar. (**A**) control, dose 0; (**B**) 410 mg dm^−3^ of K.

The highest potassium (K) doses evaluated resulted in a marked reduction in leaf spot severity, expressed as decreased necrotic lesions and canopy discoloration. This response is physiologically consistent with improved plant metabolic balance promoted by adequate K nutrition (Figure 1 and Figure 2). Potassium plays a central role in carbohydrate metabolism, enzyme activation, osmoregulation, and translocation of assimilates, promoting the synthesis of high molecular weight compounds such as proteins, starch, and structural carbohydrates while reducing the accumulation of soluble sugars, amino acids, and organic acids that often favor pathogen development [9,11,20]. Under K deficiency, the accumulation of soluble compounds in plant tissues may favor pathogen growth, increasing disease susceptibility [11].

These findings are consistent with the synthesis presented by Tripathi et al. [11], who reported that K fertilization reduces the severity of most fungal and bacterial diseases, whereas viral diseases tend to show more variable responses. In this context, the contrasting behavior observed between leaf spot and mosaic reinforces that virus–host interactions respond differently to nutritional modulation [14].

**Figure 3:**
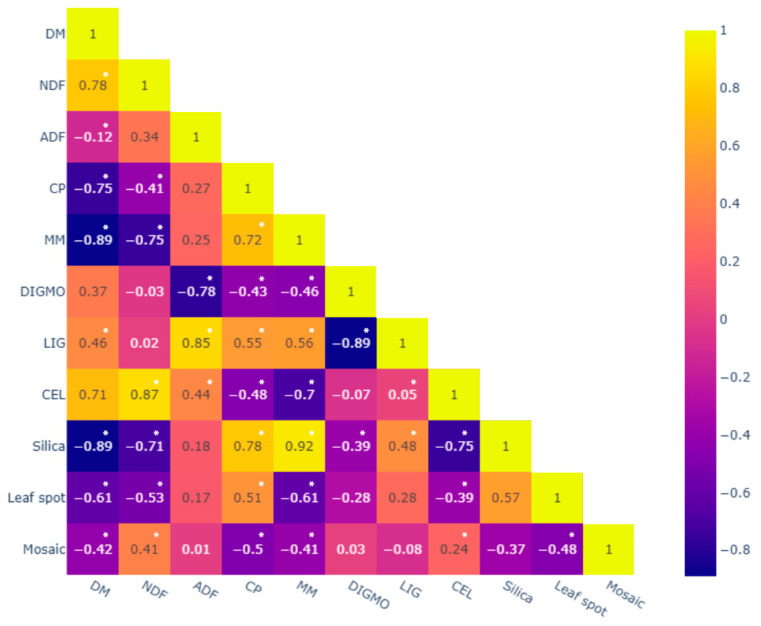
Pearson correlations between variations in chemical composition and severity of leaf spot complex (*Bipolaris maydis* and *B*. *yamadae*) and mosaic (*Johnsongrass mosaic virus*) in *Panicum maximum* cultivars. Asterisks indicate significance at *p* < 0.05 (*); DM = dry matter; NDF = neutral detergent fiber; ADF = acid detergent fiber; CP = crude protein; MM = mineral matter; DIGMO = digestibility of organic matter; LIG = lignin; CEL = cellulose; leaf spot = leaf spot complex.

Fungal pathogens responsible for leaf spot complex, such as *Bipolaris maydis* and *B. yamadae*, degrade plant cell walls through secretion of extracellular enzymes that break down cellulose, hemicellulose, and lignin, ultimately reducing photosynthetically active tissue [21]. This mechanism explains the observed negative correlations between disease severity and DM, NDF, MM, and cellulose contents (Figure 3). Nevertheless, average NDF values remained within acceptable limits for fertilized tropical grasses, suggesting that structural carbohydrate accumulation induced by enhanced growth under adequate K supply partially offsets pathogen-induced degradation [22,23]. Potassium fertilization commonly stimulates rapid leaf expansion and biomass production, increasing cell wall deposition and potentially diluting the proportional impact of tissue degradation caused by infection [11].

A positive association between leaf spot severity and crude protein (CP) content was also observed (Figure 3). This suggests that, despite infection, forage protein levels were not negatively affected, possibly because adequate K nutrition improves uptake and utilization efficiency of nitrogen and other nutrients, sustaining protein synthesis even under pathogen pressure [24]. Furthermore, pathogen-induced metabolic changes often alter carbon allocation more than nitrogen metabolism, explaining maintenance of CP levels despite tissue damage [24].

Genotypic variability played a decisive role in disease expression. The Tanzânia cultivar exhibited greater resistance to the leaf spot complex, corroborating previous reports for apomictic hybrids [3]. However, earlier studies have described higher susceptibility of this cultivar to *B. maydis* [21,25,26], indicating that disease expression depends on interactions among genotype, environmental conditions, nutritional status, and pathogen pressure [27].

In contrast to the response observed for fungal diseases, mosaic severity showed a distinct pattern in relation to potassium supply. In the Tanzânia cultivar, mosaic severity increased with increasing K doses, indicating a different physiological interaction between virus infection and host nutrition. Viral diseases are often closely linked to host metabolic activity and cellular replication processes; therefore, the enhanced plant growth promoted by fertilization may inadvertently favor viral replication and systemic movement [11,14]. For the other cultivars, however, mosaic severity followed a quadratic response, with reductions occurring beyond critical K levels. This suggests that, under adequate nutritional conditions, potassium may contribute more to increased plant tolerance than to direct resistance against viral infection.

The high mosaic severity observed in Tanzânia indicates greater susceptibility to Johnsongrass mosaic virus compared with the other cultivars. Negative correlations between mosaic severity and mineral contents suggest that infected tissues may experience nutrient redistribution or depletion due to metabolic reallocation toward defense compound synthesis or viral replication processes [21]. Similar responses have been described in other grass–virus interactions, where infection alters nutrient partitioning and carbohydrate metabolism, affecting forage quality and productivity [14].

Another important aspect concerns the relationship between nutritional quality and pathogen attraction. Forage tissues with higher CP levels can become more attractive to herbivores and potentially to vectors responsible for virus transmission, indirectly increasing disease incidence. Therefore, improvements in nutritional quality may not always correspond to lower disease occurrence, particularly for vector-transmitted viral diseases [14].

Overall, potassium nutrition plays a key role in disease dynamics in forage systems, particularly by reducing fungal disease severity. However, its effects on viral diseases are more complex and genotype-dependent. These findings highlight the importance of integrating fertilization strategies with cultivar selection and disease monitoring for effective management [27].

## 4. Materials and Methods

The experiment was carried out in the greenhouse of EMBRAPA Gado de Corte (20°26′48″ S 54°43′07″ W, 538 m above sea level), located in Campo Grande—Mato Grosso do Sul, from June 2019 to January 2020, totalling 164 days. A duration is defined to avoid root restriction associated with limited soil volume in pot studies, which could interfere with treatment responses.

The experimental soil was classified as a typical Quartzarene Neosol (RQo), according to the Brazilian Soil Classification System [28], collected in Campo Grande—MS, in the Córrego do Guariroba basin (20°33′41″ S, 54°22′30″ W, 501 m above sea level). The collection was carried out in a layer 0-20 cm deep, passed through a sieve with a 4 mm mesh, air-dried (Terra Fine Dried in Air—TFSA), and again passed through a sieve with a 2 mm mesh. Samples were collected for chemical analysis before fertilization (Table 3).

The experimental soil, classified as a Quartzarenic Neosol, is naturally characterized by low fertility, low cation exchange capacity, and reduced potassium availability due to its sandy texture and high susceptibility to nutrient leaching. Prior to fertilization, soil acidity was corrected by liming, increasing the pH (CaCl_2_) from 4.34 to 5.79, a range considered adequate for the growth of Megathyrsus maximus cultivars under tropical conditions. Before sowing, implantation fertilization was carried out with 54.68 mg dm^−3^ of phosphorus (P); 1389 mg dm^−3^ of dolomitic limestone; 67.63 mg dm^−3^ of sulfur (S); 11.02 mg dm^−3^ of zinc (Zn); 11.02 mg dm^−3^ of copper (Cu); 2.76 mg dm^−3^ of boron (B); and 1.37 mg dm^−3^ of molybdenum (Mo). After fertilization, the soil was incubated for 40 days close to field capacity to evaluate soil nutrients. Each experimental unit was supplied with a pot containing 2.55 dm^−3^ of soil, duly identified, where 50 seeds were sown. After 15 days of sowing, thinning was carried out, leaving six plants per pot.

The experiment was designed in randomized blocks in a factorial scheme, with six cultivars of *Panicum maximum* (cvs. Tanzânia, Quênia, Mombaça, Zuri, Massai and Tamani) and four doses of K (0, 205, 410 and 820 mg dm^−3^) in the form of potassium chloride (KCl; 58% K_2_O), with three repetitions.

Every 28 days, all forage from each experimental unit was cut at pre-established heights, totaling five evaluative cuts. The smaller cultivars of *Panicum maximum* (cvs. Massai and Tamani) were cut to 15 cm and the others (cvs. Tanzânia, Quênia, Mombaça, and Zuri) to 20 cm of residue. This material was packed in paper bags, dried, ground, and sent to determine the chemical composition. The interval between courts was considered the 28-day review cycle.

Potassium fertilization was divided into five equal applications, carried out after each cutting. Thus, the K doses per application corresponded to 0, 41, 82, and 164 mg dm^−3^, ensuring a gradual supply of nutrients throughout the experimental period. The same fertilization protocol was applied to all cultivars. Fertilization with KCl was carried out after each cutting, diluted in water, and applied to the soil using a graduated pipette in milliliters, according to the proposed treatments. Nitrogen fertilization was the same for all treatments and corresponded to 100 mg dm^−3^ of N, in the form of urea (46% N).

**Figure 4:**
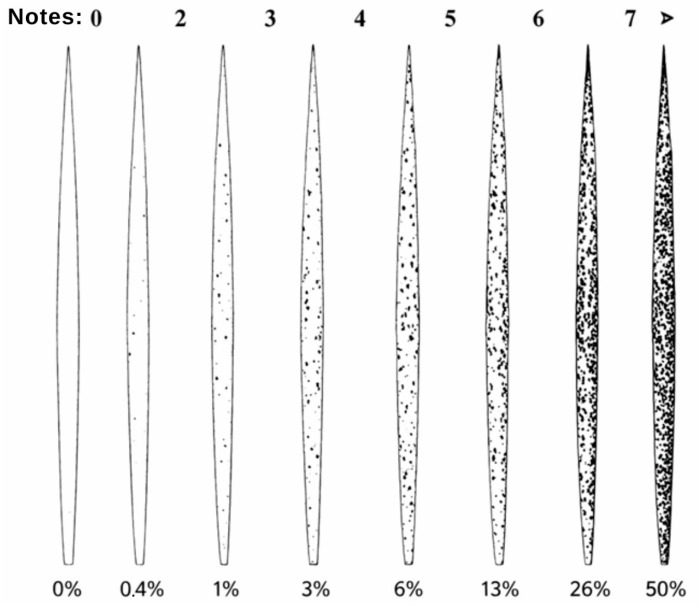
The diagrammatic scale used to evaluate the severity of the leaf spot complex, caused by *Bipolaris maydis* and *B. yamadae*, in *Panicum maximum*, proposed by Martinez, Franzener, and Stangarlin and modified by Fernandes et al.

To assess the severity of the leaf spot complex, the diagrammatic scale developed by Martinez, Franzener, and Stangarlin [29] and modified by Fernandes et al. [30] is illustrated in Figure 4.

Quantification of the severity of symptoms caused by the mosaic (*Johnsongrass mosaic virus*) was done based on a descriptive key (Table 4), previously prepared with five levels, expressed in notes.

Three assessments of the diseases, which occurred due to natural infection, were carried out immediately before cuts 2, 3, and 4. Using the severity data over time, the area under the disease progress curve (AACPD) was calculated for all diseases. Evaluated diseases [29] are expressed by the formula:

AACPD = ∑n − 1 [(Xi + Xi + 1)] × 0.5] × [ti + 1 − ti]

where n is the number of evaluations, x is the severity of the disease, and [ti + 1 − ti] is the interval of consecutive evaluations.

The whole plant samples, cut and packed in paper bags, were dried in forced air circulation ovens at 55 °C until a constant weight was obtained, and then they were ground in a Willey knife mill with a 1 mm sieve. In the laboratory, dry matter—DM, crude protein content—CP, neutral detergent fiber—NDF, acid detergent fiber—ADF, mineral matter—MM, digestibility of organic matter—DIGOM, and lignin content were quantified—LIG, while silica and cellulose were evaluated using the near-infrared light reflectance spectroscopy (NIRS) system, according to the procedures described by Marten et al. [30]. For these analyses, NIRS accuracy curves were made using model 5000 software (FOSS, Hilleroed, Denmark) type V1.02.

Correlations were obtained by Pearson’s correlation analysis and a *t*-test, considering significance at *p* ≤ 0.05. The demonstration coefficient classification was r greater than 70%, meaning a strong association, and moderate when r was less than or equal to 70% and greater than 30%.

The data obtained from disease assessment were subjected to variance and regression analyses. The mathematical model contained the fixed effects of cultivars, potassium doses, and the interaction between them. The effects of the cultivars were evaluated using the Tukey test with a significance of 5%. The effects of K doses were studied by regression analysis. The linear and quadratic models were tested, and the model was selected according to the significance of the regression coefficients, adopting a 5% probability level and the coefficient of determination (R2).

## 5. Conclusions

Increasing doses of potassium reduced the severity of leaf spot complex and increased that of mosaic in *Panicum maximum* cultivars. The occurrence of the leaf spot complex did not affect the nutritional value of the evaluated cultivars, regardless of the severity level. However, the mosaic virus elevated the fraction of structural carbohydrates in the plants. The Tânzania cultivar exhibited resistance to the leaf spot complex but susceptibility to the mosaic virus. The dose of 410 mg K dm^−3^ was sufficient for the significant decrease in leaf spot severity.

## Figures and Tables

**Table 1:** Descriptive statistics of the parameters observed in the chemical composition and severity of the leaf spot complex (*Bipolaris maydis* and *B. yamadae*) and the mosaic (*Johnsongrass mosaic virus*) in different cultivars of *Panicum maximum*.

Variables	N	Mean ± SD	Minimum	Maximum	CV (%)
Leaf spot complex	79	26.72 ± 26.01	5.60	76.93	97.36
Mosaic	71	69.52 ± 10.12	56.28	98.17	14.56
Dry matter (%)	72	91.65 ± 1.29	89.52	93.20	0.89
Mineral matter (%)	72	5.56 ± 1.29	3.48	8.91	23.24
Crude protein (%)	72	7.61 ± 1.61	5.13	12.10	21.25
NDF (%)	72	71.18 ± 1.61	62.93	80.57	6.13
ADF (%)	72	38.93 ± 2.09	34.50	44.73	5.38
DIGMO (%)	72	43.95 ± 5.26	35.10	55.49	11.97
Lignin (%)	72	3.52 ± 0.55	2.30	4.90	15.66
Cellulose (%)	72	33.95 ± 1.94	27.52	38.75	5.73
Silica (%)	72	1.61 ± 1.03	0.00	4.44	63.91

N = number of observations; SD = standard deviation; CV = coefficient of variation; NDF = neutral detergent fiber; ADF = acid detergent fiber; DIGMO = digestibility of organic matter.

**Table 2:** Complex severity of leaf spots (*Bipolaris maydis* and *B. yamadae*) and mosaic (*Johnsongrass mosaic virus*) in *Panicum maximum* cultivars in response to different doses of potassium expressed as area under the disease progress curve (AACPD).

Cultivars	Doses of K (mg dm^−3^)				Equation	R^2^
	0	205	410	820		
	**Leaf Spot Complex**					
Tanzânia	36.53 c	5.60 c	5.60 a	5.60 a	Ŷ = 24.160 − 0.150 K	46.67
Quênia	50.20 b	24.05 b	5.60 a	5.60 a	Ŷ = 39.742 − 0.256 K	72.24
Mombaça	54.62 b	71.28 a	5.60 a	5.60 a	Ŷ = 61.284 − 0.376 K	60.80
Zuri	60.92 ab	5.60 c	5.60 a	5.60 a	Ŷ = 38.794 − 0.269 K	46.67
Massai	69.18 a	22.12 b	5.60 a	5.60 a	Ŷ = 50.361 − 0.344 K	64.45
Tamani	58.23 ab	27.01 b	5.60 a	5.60 a	Ŷ = 59.276 − 0.254 K	34.14
	**Mosaic**					
Tanzânia	62.06 a	78.98 a	83.72 a	91.93 a	Ŷ = 67.186 − 0.0334 K	86.18
Quênia	58.20 a	67.74 a	65.91 b	68.87 b	Ŷ = 59.311 + 0.0318 K − 0.00003 K^2^	78.28
Mombaça	67.74 a	71.60 a	72.28 ab	60.13 b	Ŷ = 67.632 + 0.0307 K − 0.00005 K^2^	99.85
Zuri	67.74 a	77.50 a	74.77 ab	72.16 b	Ŷ = 68.84 + 0.0361 K − 0.00004 K^2^	71.44
Massai	58.20 a	68.87 a	76.58 ab	60.13 b	Ŷ = 57.573 + 0.0832 K − 0.0001 K^2^	97.77
Tamani	58.20 a	72.72 a	65.02 b	67.74 b	Ŷ = 60.512 + 0.0367 K − 0.00004 K^2^	40.58

Means followed by distinct lowercase letters in the column differ by Tukey’s test (*p* < 0.05).

**Table 3:** Chemical properties of the soil at the beginning of the experimental period in the 0 to 20 cm layer.

Soil	pH	Ca^2+^	Mg^2+^	K^+^	Al^3+^	H + Al	SB	PCEC	ECEC	BS	m	OM	P
RQo	CaCl^2^	cmol_c_dm^−3^								%			mg.dm^−3^
	5.79	1.70	1.07	0.15	0.00	1.15	2.92	4.07	2.92	71.7	0.00	1.46	44.6

SB: sum of bases (Ca + Mg + K); PCEC: potential cation exchange capacity (H + Al + Ca + Mg + K); ECEC: effective cation exchange capacity (Ca + Mg + K + Al); BS: base saturation (SB/PCEC) × 100; m: aluminum saturation [Al/PCEC] × 100, where P and K—Mehlich I; OM—modified South Dakota; H + Al—SMP buffer; Ca and Mg—Mehlich III.

**Table 4:** A descriptive key was used to evaluate the severity of the mosaic (*Johnsongrass mosaic virus*) in leaves of *Panicum maximum* cultivars.

Scale	Description
1	Plants without apparent symptoms (although they may contain viruses in a latent state) appear healthy, without discolouration, tissue deformation, or dwarfism.
2	Plants with mild mosaic or yellowing symptoms, without deformation or dwarfism.
3	Plants with strong mosaic symptoms, moderate deformation, and mild stunting.
4	Plants with intense mosaic and/or tissue necrosis, very pronounced deformation of organs, and strong dwarfism.
5	All very strong symptoms with necrosis and death of the plant in more advanced stages.

## Data Availability

The data generated and analyzed during this study are fully included within this published article.

## Abbreviations

The following abbreviations are used in this manuscript:

AACPD	Area Under the Disease Progress Curve
ADF	Acid Detergent Fiber
BRS	An acronym for cultivars registered in Brazil
BS	Base Saturation
Ca	Calcium
CAPES	Coordination for the Improvement of Higher Education Personnel
CEL	Cellulose
CNPq	National Council for Scientific and Technological Development
CP	Crude Protein
CV	Coefficient of Variation
cv./cvs.	Cultivar/Cultivars
DIGOM	Digestibility of Organic Matter
DM	Dry Matter
ECEC	Effective Cation Exchange Capacity
EMBRAPA	Brazilian Agricultural Research Corporation
FUNDECT	Foundation for the Support of the Development of Education, Science, and Technology in the State of Mato Grosso do Sul
H + Al	Potential Acidity
JGMV	Johnsongrass Mosaic Virus
K	Potassium
K_2_O	Potassium Oxide
KCl	Potassium Chloride
LIG	Lignin
Mg	Magnesium
MM	Mineral Matter
Mo	Molybdenum
MS	Mato Grosso do Sul (Brazilian state)
N	Nitrogen
NDF	Neutral Detergent Fiber
NIRS	Near-Infrared Reflectance Spectroscopy
OM	Organic Matter
P	Phosphorus
PCEC	Potential Cation Exchange Capacity
R^2^	Coefficient of Determination
RQo	Quartzarene Neosol soil classification code
SD	Standard deviation of the mean
SMP	Shoemaker, McLean, and Pratt buffer method
TFSA	Terra Fine Dried in Air
